# The PI3K-Akt pathway is a multifaceted regulator of the macrophage response to diverse group B *Streptococcus* isolates

**DOI:** 10.3389/fcimb.2023.1258275

**Published:** 2023-10-19

**Authors:** Yadira S. De-Leon-Lopez, Michelle E. Thompson, Jessica J. Kean, Rebecca A. Flaherty

**Affiliations:** Department of Biology and Health Science, Aquinas College, Grand Rapids, MI, United States

**Keywords:** group B streptococcus, macrophage, PI3K-Akt pathway, phagocytosis, cell death, inflammatory response, intracellular signaling

## Abstract

Group B *Streptococcus* (GBS), also known as *Streptococcus agalactiae*, is a common member of the microbial flora in healthy individuals. However, problems may arise when GBS-colonized mothers become pregnant. GBS may be transferred from a colonized mother to her newborn or developing fetus, which may result in complications such as miscarriage, pre-term birth, meningitis, pneumonia, or sepsis. Macrophages play an especially important role in the fetal and newborn response to GBS due to the limited development of the adaptive immune system early in life. The goal of this study was to expand what is currently known about how GBS manipulates macrophage cell signaling to evade the immune system and cause disease. To this end, we investigated whether the PI3K-Akt pathway was involved in several key aspects of the macrophage response to GBS. We explored whether certain GBS strains, such as sequence type (ST)-17 strains, rely on this pathway for the more rapid macrophage uptake they induce compared to other GBS strains. Our findings suggest that this pathway is, indeed, important for macrophage uptake of GBS. Consistent with these findings, we used immunofluorescence microscopy to demonstrate that more virulent strains of GBS induce more actin projections in macrophages than less virulent strains. Additionally, we explored whether PI3K-Akt signaling impacted the ability of GBS to survive within macrophages after phagocytosis and whether this pathway influenced the survival rate of macrophages themselves following GBS infection. The PI3K-Akt pathway was found to promote the survival of both macrophages and intracellular GBS following infection. We also observed that inhibition of the PI3K-Akt pathway significantly reduced GBS-mediated activation of NFκB, which is a key regulator of cell survival and inflammatory responses. Overall, these insights into strain-dependent GBS-mediated manipulation of the PI3K-Akt pathway and its downstream targets in infected macrophages may provide new insights for the development of diagnostic and therapeutic tools to combat severe GBS disease.

## Introduction

Group B *Streptococcus* (GBS), also known as *Streptococcus agalactiae*, is an opportunistic bacterial pathogen that is frequently part of a healthy individual’s microbial flora (Doran and Nizet, 2004; Horvath et al., 2014; Korir et al., 2017b). However, while severe infections in healthy adults are uncommon, GBS is a leading cause of neonatal infections and a key causative agent of pregnancy complications related to microbial infection (Doran and Nizet, 2004; Manning et al., 2008; Manning et al., 2009; Horvath et al., 2014; Korir et al., 2017b). Epidemiological data indicates that not all GBS strains are equally virulent; there are certain sequence types (ST) of GBS that are more closely associated with causing serious pregnancy and post-delivery complications for GBS-colonized women and their babies (Manning et al., 2008; Manning et al., 2009). Though any sequence type of GBS could potentially cause a serious infection, infections with more virulent STs, such as ST-17 and ST-19 strains, more frequently lead to preterm birth, stillbirth, neonatal sepsis, pneumonia, lung injury, and neonatal meningitis (Doran and Nizet, 2004; Manning et al., 2008; Manning et al., 2009; Horvath et al., 2014; Nan et al., 2015; Wortham et al., 2016; Korir et al., 2017b).

Because of their underdeveloped adaptive immune systems, developing fetuses and newborns rely heavily upon their innate immune systems, of which macrophages are a major component (Basha et al., 2014; Kumar and Bhat, 2016; Korir et al., 2017b). An understanding of the interaction of GBS and macrophages is, therefore, an important research area to explore. Prior studies from our group and our collaborators have shown that particularly virulent GBS strains, such as ST-17 strains, are engulfed by macrophages at a higher rate and survive in macrophages longer than other sequence types (Korir et al., 2017a; Flaherty et al., 2021). ST-17 strains also induce a greater degree of macrophage cell death following GBS uptake than other STs (Flaherty et al., 2021). These differences in the host response to genetically distinct GBS strains may help to at least partially explain the differences in virulence that have been observed epidemiologically.

Previously, we and our collaborators performed an antibody array in which we identified numerous signaling pathways that were altered in macrophages in response to GBS infection (Flaherty et al., 2021). In that study, we identified changes in many members of the PI3K-Akt pathway in response to a diverse set of GBS isolates, but the physiological implications of these changes were not explored at that time (Flaherty et al., 2021). The PI3K-Akt pathway is known to be involved with several key responses in macrophages, such as cytoskeletal rearrangements, regulation of phagocytosis, regulation of cell survival and cell death, and induction of inflammatory responses (Song et al., 2005; Vergadi et al., 2017). With this information in mind, we hypothesized that there was a connection between GBS-mediated regulation of the PI3K-Akt pathway and key aspects of pathogenesis such as phagocytic uptake, viability of GBS within macrophages, viability of macrophages post-infection, and initiation of the inflammatory response.

To address this hypothesis, we first used fifteen previously described GBS strains obtained from either GBS-colonized mothers or from infants who were infected with GBS to confirm that PI3K-Akt signaling is activated in response to at least some GBS stains (Manning et al., 2008; Manning et al., 2009; Flaherty et al., 2019a; Flaherty et al., 2021). The strains selected represent four distinct sequence types (ST-17, ST-19, ST-12, and ST-1) and three distinct capsule types (CPS III, CPS-II, and CPS-V) (Manning et al., 2008; Manning et al., 2009). Our follow-up experiments to assess the physiological implications of PI3K-Akt activation in response to GBS focused primarily on four of these representative strains, which had all been isolated from colonized mothers: GB00112 (GB112, ST-17, CPS III), GB00590 (GB590, ST- 19, CPS III), GB00653 (GB653, ST-12, CPS II), and GB00020 (GB20, ST-1, CPS V) (Manning et al., 2008).

In the present study, we sought to use these isolates to expand the current understanding regarding how GBS manipulates macrophage cell signaling to evade the innate immune response and cause disease. Specifically, we utilized Western Blotting, colony counting-based phagocytic uptake and intracellular survival assays, cytotoxicity assays, and immunofluorescence microscopy to investigate whether the PI3K-Akt pathway is involved in several key aspects of the macrophage response to GBS. We successfully confirmed the activation of this pathway in response to certain GBS sequence types, and we explored whether particular GBS strains, such as ST-17 and ST-19 strains, rely on this pathway for the more rapid macrophage uptake they induce compared to other less virulent GBS strains. Our findings indicated that GBS uptake by macrophages involves actin-mediated cytoskeletal rearrangements, and that PI3K-Akt signaling does play a significant role in phagocytosis of these GBS strains by macrophages. Additionally, we evaluated whether PI3K-Akt signaling impacts the ability of GBS to survive within macrophages after phagocytosis and whether this pathway influences the survival rate of macrophages themselves following GBS infection. The PI3K-Akt pathway was found to promote the survival of both macrophages and GBS following infection. Furthermore, inhibition of the PI3K-Akt pathway significantly reduced GBS-mediated activation of NFκB, which is a key regulator of cell survival and inflammatory responses (D’Acquisto et al., 2002; Ghosh et al., 2003). Overall, these insights into GBS-mediated manipulation of the PI3K-Akt pathway and its downstream targets in infected macrophages may provide new insights for the development of diagnostic and therapeutic tools to combat severe GBS disease.

## Materials and methods

### Bacterial strains

This investigation included a total of 15 GBS strains obtained from either GBS-colonized mothers or from infants with severe GBS disease (Manning et al., 2008; Manning et al., 2009). These strains represent four distinct sequence types (ST-17, ST-19, ST-12, and ST-1) and three distinct capsule types (CPS III, CPS-II, and CPS-V) (Manning et al., 2008; Manning et al., 2009). Most experiments focused primarily on four of these representative strains, which had all been isolated from colonized mothers: GB00112 (GB112, ST-17, CPS III), GB00590 (GB590, ST- 19, CPS III), GB00653 (GB653, ST-12, CPS II), and GB00020 (GB20, ST-1, CPS V) (Manning et al., 2008). The major characteristics of all 15 strains are summarized in **Table 1**.

**Table 1:** GBS Strain Information.

GB#	ST	CPS	Clinical Type*
GB112	17	III	colonizing
GB411	17	III	invasive
GB97	17	III	colonizing
GB418	17	III	invasive
GB590	19	III	colonizing
GB571	19	III	colonizing
GB36	19	III	invasive
GB79	19	III	invasive
GB653	12	II	colonizing
GB285	12	II	colonizing
GB910	12	II	invasive
GB1455	12	II	invasive
GB37	1	V	invasive
GB20	1	V	colonizing
GB310	1	V	invasive

* Strains were isolated from colonized mothers during prenatal screening (Manning et al., 2008) or from symptomatic neonates with invasive disease (Manning et al., 2009). Strains collected during routine prenatal screening may still be capable of causing invasive infection.

Prior to infection, the selected strains were grown in Todd-Hewitt broth (THB) at 37°C for 16 to 20 hours. Next, a sample of each overnight culture was subcultured into fresh THB and incubated for approximately 2 hours at 37°C to obtain log phase cultures (optical density at 600 nm of approximately 0.4). Lastly, the bacteria from the log phase cultures were washed with sterile phosphate-buffered saline (PBS) and resuspended in RPMI 1640 (ATCC) at a concentration of 4x10^7^ CFU/mL.

### THP-1 cell culture and infection

THP-1 monocyte-like cells (ATCC TIB-202) were cultured at a temperature of 37°C with 5% CO_2_. Cells were suspended in RPMI 1640 media (Gibco) supplemented with 10% fetal bovine serum (FBS; ATCC), and 1% penicillin/streptomycin (Gibco). Prior to plating for infection experiments the THP-1 cells were suspended in RPMI 1640 with 2% FBS, 1% penicillin/streptomycin, and 100nM phorbol 12-myristate 13 acetate (PMA; Sigma-Aldrich) at a concentration of 2x10^6^ cells/mL to differentiate the monocytes into macrophages as described previously (Flaherty et al., 2019a; Flaherty et al., 2021). The THP-1 cells were plated into 6-well plates (CytoOne) at a density of 4x10^6^ cells per well (using 2 mL of the 2x10^6^ cells/mL cell suspension) or into 24-well plates (CytoOne) at a density of 1x10^6^ cells per well (using 0.5 mL of the cell suspension) and incubated for 24-48 hours.

PMA differentiation has been reported to generate cells that have similar properties to peripheral blood mononuclear cell monocyte-derived macrophages (Starr et al., 2018). When used at similar concentrations and incubation times as those utilized here, PMA induces THP-1 cells to become larger, adherent, CD11 and CD14 positive, and also to have enhanced mRNA expression of genes relating to cellular communication and cytokine regulation, such as IL-1 beta and IL-8 (Schwende et al., 1996; Korir et al., 2017a; Starr et al., 2018; Liu et al., 2023). Though specific macrophage markers were not assessed here following PMA differentiation, cytokine profiles of infected and control PMA-differentiated THP-1 cells are available in our prior work (Flaherty et al., 2019a). We anticipate these studies represent a simplified model to assess M1-type macrophage responses to GBS.

Immediately prior to the start of an infection experiment, the macrophages were washed twice with PBS, given fresh RPMI media (with no FBS or antibiotic supplements) and incubated for 1-2 hours. In cases where small molecule inhibitors and vehicle controls were to be used, they were applied at this time. The cells were then infected with the desired GBS strain at a multiplicity of infection (MOI) of 10 bacteria per host cell (4x10^7^ CFU per well of a 6 well plate; 1x10^7^ CFU per well of a 24 well plate). After incubating for 1 hour at 37°C with 5% CO_2_, the cell culture media was aspirated and the cells were washed with PBS to remove bacteria that had not been engulfed. Then RPMI 1640 with 2% FBS containing 100 µg/ml gentamicin (Gibco) and 5µg/ml penicillin (Sigma) was added to kill any remaining extracellular bacteria. After an additional incubation period of 1-48 hours (as indicated for each experiment), the cells were washed with PBS and prepared for the next stage of analysis.

### SDS-PAGE and Western Blotting

For samples to be analyzed by Western Blotting, the cells were infected as detailed above, washed with PBS, and lysed with lysis buffer as described previously (Flaherty et al., 2021). To determine the protein concentration of the lysate samples, a bicinchoninic acid assay (BCA, Thermo-Fisher Scientific) with bovine serum albumin (BSA) protein standards was used. The normalized lysate samples were loaded onto hand cast 10% polyacrylamide gels (BioRad) and allowed to separate (50mA for approximately 1 hour). The protein samples were transferred to a polyvinylidene difluoride (PVDF) membrane (25v for 1-2 hours, followed by 75v for an additional 1-2 hours), blocked in tris-buffered saline with 1% Tween-20 (TBST) and 5% BSA for 2 hours at room temperature, and incubated with primary antibodies (1:1000 dilution in blocking solution; overnight at 4°C or 2 hours at room temperature). The blots were again washed in TBST and then incubated with secondary antibodies (1:2500-1:5000 dilution in blocking solution; 1-2 hours at room temperature). The blots received a final series of washes in TBST prior to detection of the proteins with either a colorimetric TMB substrate reagent (1 STEP Ultra TMB Botting Solution, Thermo-Fisher Scientific) or SuperSignal West Pico PLUS Chemiluminescent Substrate (Thermo-Fisher Scientific), according to the manufacturer’s instructions. The chemiluminescent blots were imaged with a digital Amersham ImageQuant800 imager. Differences in protein abundance and activity were determined by densitometry using ImageJ. Beta-actin and beta-tubulin were used as loading controls.

### Immunofluorescence microscopy staining and imaging

THP-1 cells were seeded onto glass coverslips which had been placed in 6 well plates, and they were differentiated using the PMA differentiation protocol described above. The macrophages were then infected with GBS as described above. Following infection, they were fixed with 4% paraformaldehyde (PFA) in PBS (10 minutes at room temperature, followed by overnight incubation at 4°C). The fixed cells were washed with PBS and placed in IFM blocking solution (PBS with 1% (w/v) normal goat serum, 2% (v/v) Triton, and 0.5% (v/v) Tween 20) for 2 hours at room temperature. After the blocking solution was removed, the cells were incubated with primary antibodies for 16 to 20 hours (1:400 in blocking solution). Then, the cells were washed with PBS and incubated with secondary antibodies (1:200 in blocking solution) for 2 hours at room temperature. Lastly, after washing again with PBS, the cells were stained with DAPI (1:500 in blocking solution) and mounted on glass slides with Fluoromount G (Thermo-Fisher Scientific).

Images were captured utilizing fluorescence microscopy (Leica DMIL LED fluorescence microscope with Ocular software (version 2); 20x, 40x, or 60x objectives were used, depending on the specific experiment). Each experimental condition was tested in biological triplicate (3 independent wells), at minimum, in 6-well plates. At least three fields per well were captured for a minimum of 5,500 cells counted per condition for the NF kappa B visualization and at least 1,500 cells counted per condition for the actin visualization experiments.

### Antibodies and stains

Primary antibodies used for immunofluorescence microscopy and Western Blotting were obtained from Cell Signaling Technology and included: Beta-actin (#3700 and #4970), phospho-p70s6k T389 (#9234), NF kappa B p65 (#8242S), beta-tubulin (#2128S), Caspase-3 (#14220), Caspase-1 (#3866), Phospho-MLKL (#91689). Secondary antibodies used to detect the primary antibodies for Western Blotting were obtained from Thermo Fisher Scientific, and included goat anti-rabbit and goat anti-mouse IgG-HRP (#31460 and #31430). Secondary antibodies used for immunofluorescence microscopy were obtained from Molecular Probes (Life Technologies), and included goat anti-rabbit IgG AlexaFluor488 and goat anti-mouse IgG AlexaFluor594.

### Vehicle controls and chemical inhibitors

For the infection experiments in which the PI3K-Akt pathway or cell death proteins were inhibited, dimethyl sulfoxide (DMSO; from ATCC) was used as a vehicle control at a final concentration of 0.5% in cell culture media. LY294002 (LY; #9901 from Cell Signaling Technology) was used to inhibit the PI3K-Akt pathway at a final concentration of 50 µM, and it was added to the cells 1-2 hours before the infection. LY294002 has been demonstrated previously to function as a highly selective inhibitor of PI3K. When it is used at a concentration of 50 μM with a pre-treatment period of at least 1 hour (as it was in our studies), it is able to specifically abolish PI3K activity (IC50 = 0.43 μg/ml; 1.40 μM) (Vlahos et al., 1994). Of note, it does not inhibit other similar lipid and protein kinases, including PI4K, c-Src, MAPK, and PKC (Vlahos et al., 1994). Z-VAD-fmk (ApexBio) was used as a pan caspase inhibitor at a final concentration of 50 µM, and it was also added to cells 1-2 hours before the infection.

### Ethidium homodimer cell death assay

To measure the survival rate of macrophages 24 or 48 hours after the initial infection period, an ethidium homodimer-1 (Thermo Fisher Scientific) membrane permeabilization assay was used. After the macrophages were infected with GBS (MOI 10 for 1 hour) as before, they were washed and treated with 100µg/mL gentamicin and 5µg/mL penicillin G for 24-48 hours, as indicated for each experiment. They were then incubated with ethidium homodimer-1 (4 µM) in PBS, which is a fluorescent dye that enters dead cells by crossing their damaged membranes and then binding tightly to DNA. Macrophages were visualized by fluorescence microscopy (Leica DMIL LED fluorescence microscope with Ocular software (version 2); 20x or 40x objective, as indicated) to distinguish dead macrophages (bright red) from living macrophages (unstained). Images were captured from at least 6 biological replicates per condition, with at least 3 fields captured per well. At least 9,000 cells were counted per condition.

### GBS uptake and survival assays in macrophages

THP-1 cells were infected with GBS at an MOI of 10 for 1 hour as described above following an initial treatment with either DMSO or LY294002. Throughout the experiment, the cells were incubated at 37°C with 5% CO_2_. A final GBS inoculum sample was collected from each biological replicate at the end of the 1 hour infection period. These collected bacteria were serially diluted and subsequently plated in triplicate on Todd-Hewitt agar. The plates were incubated overnight at 37°C to quantify the CFUs from each well.

After collecting the final inoculum samples, the THP-1 cells were washed with PBS and incubated with antibiotics (100µg/mL gentamicin and 5µg/mL penicillin G). Then, 1 hour post-antibiotics, the cells were again washed and then lysed with 0.1% Triton X-100 in PBS (Sigma). The samples were collected and vortexed to thoroughly break up host cell membranes and release intracellular GBS into solution. The samples were then serially diluted and plated in triplicate on Todd-Hewitt agar; they were incubated overnight at 37°C to quantify the intracellular bacteria. The same procedure was utilized to quantify viable intracellular bacteria at 24 hours post-antibiotics as a means of assessing long-term survival of GBS in macrophages.

### Statistical analysis

GraphPad Prism 9 and Microsoft Excel were used to perform statistical analyses of all the experiments described. When comparing data sets with three or more groups, ANOVA was used to initially identify data sets for which significant differences were present, with p-values of less than 0.05 being considered statistically significant. Following ANOVA, data sets with significant differences were compared via Dunnett’s test (in which means from GBS infection conditions were compared to the mock infection mean) or Tukey’s test (in which the means of all experimental conditions were compared to each other). In cases where only two groups were being compared (such as DMSO *vs*. LY294002 for the same strain), t-tests were used to identify significant differences.

## Results

### Strain-dependent activation of the PI3K-Akt pathway in GBS-infected macrophages

Prior studies from our group and our collaborators using antibody microarrays demonstrated that diverse GBS strains modulate many proteins associated with the PI3K-Akt pathway during macrophage infection (Flaherty et al., 2021). As this pathway can participate in the regulation of a variety of cellular responses (Song et al., 2005; Vergadi et al., 2017), our initial analyses were not sufficient to link pathway manipulation to specific pathogenic outcomes during GBS infection of macrophages. However, we hypothesized that manipulation of this pathway by GBS would most likely impact macrophage responses related to phagocytic uptake of the bacteria as well as regulation of cell death and survival signaling within the infected macrophages (Song et al., 2005; Williams et al., 2006; Shanware et al., 2013; Vergadi et al., 2017; Lv et al., 2019).

**Figure 1:**
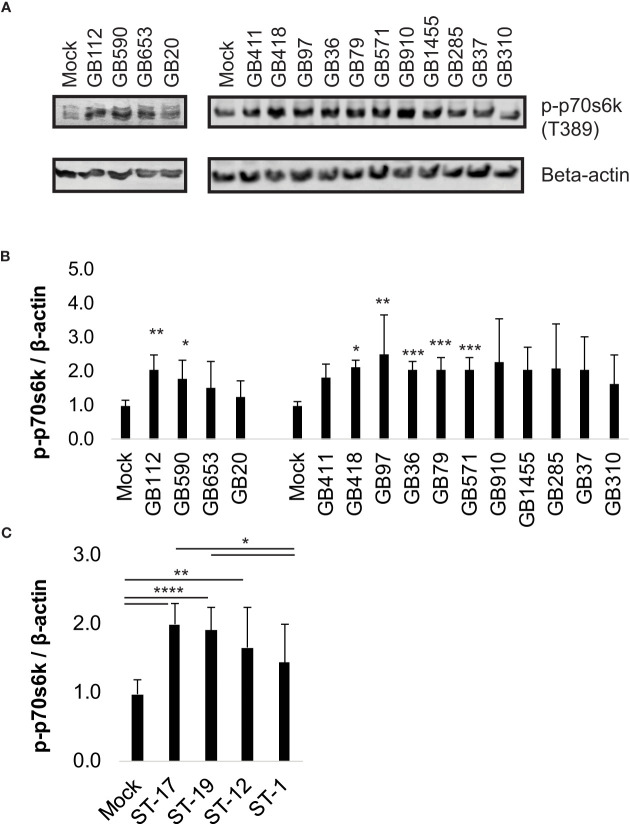
Specific GBS strains induce p70s6k activation. THP-1 macrophages were infected with one of 15 different strains of GBS at an MOI of 10 for one hour, washed, and treated with antibiotics for an additional hour prior to lysate collection. Lysates were assessed for phosphorylated (active) p70s6k **(A, B)**, and densitometry was used to compare differences between infection conditions. Representative Western blots from one biological replicate with its corresponding loading control (beta-actin) are shown **(A)**. Equal amounts of the same protein lysate preparations were loaded onto the gels for each protein. Densitometry values represent pooled results from at least three independent biological replicates, and error bars represent standard deviations of the mean **(B)**. For the full 15 strain panel (tested in two groups as shown), significance was determined by ANOVA with *post-hoc* Dunnett’s testing to compare each infection condition to the mock infection (*, p=0.01-0.05; **, p=0.001-0.01; ***, p=0.0001-0.001; ****, p<0.0001). Densitometry results from these strains were then grouped by ST to compare differences in protein activation among the groups **(C)**. When grouped by STs, significance was determined by ANOVA (p-value: <0.0001) with *post-hoc* Tukey’s testing to compare the mean of each infection condition to the mean of each of the other conditions (*, p=0.01-0.05; **, p=0.001-0.01; ***, p=0.0001-0.001; ****, p<0.0001).

We began by first selecting a well-characterized downstream target of the PI3K-Akt pathway, phospho-p70s6k (T389), to verify pathway activation at our infection conditions of interest and to ensure that the pathway would respond appropriately to manipulation by the small molecule inhibitor we planned to use in our subsequent studies (LY294002). Phospho-p70s6k had been identified by the antibody array screen as having an altered response in macrophages following GBS infection with at least some of the GBS strains tested (Flaherty et al., 2021). We assessed 15 diverse GBS strains representing four STs and three CPS types at the same infection conditions used in the array (1 hour infection at MOI=10 followed by antibiotic treatment for 1 hour prior to collecting lysates) and found that seven out of eight strains in the ST-17 and ST-19 groups (both of which are CPSIII), induced a significant increase in phospho-p70s6k compared to mock infection (**Figures 1A, B**). None of the ST-12 or ST-1 strains were found to induce p70s6k signaling changes that were statistically different from the mock infection, though all GBS strains analyzed trended toward increased activity levels of this protein (**Figures 1A, B**). When the phospho-p70s6k densitometry values induced by the individual strains were grouped by ST, the ST-17 and ST-19 groups again induced significant increases in phospho-p70s6k compared to mock infection, with the ST-17 group inducing the greatest increases in the activity of this protein on average (**Figure 1C**). When pooled together in this way, strains in the ST-12 group also induced significantly greater levels of phospho-p70s6k compared to mock infection, though the difference was not as great as the two CPSIII STs. The ST-1 group was not significantly different from the mock infection, and this group was also found to induce significantly less phospho-p70s6k than both the ST-17 and ST-19 groups.

**Figure 2:**
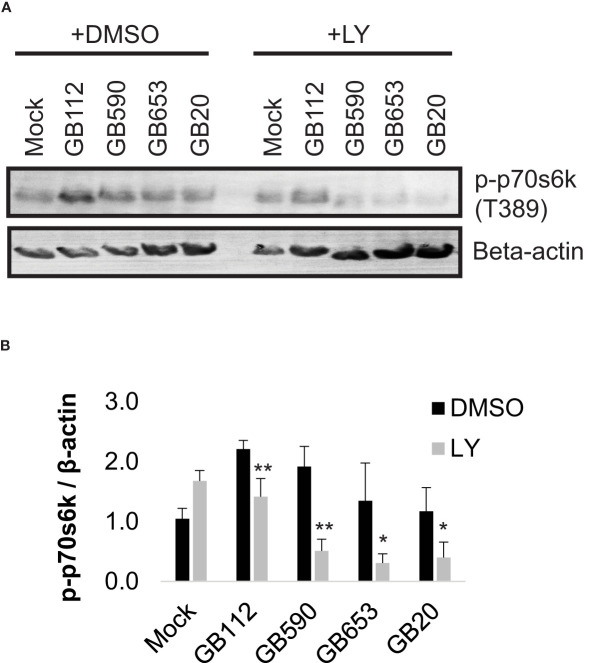
GBS induces p70s6k activation via the PI3K-Akt pathway. THP-1 macrophages were treated with DMSO or LY294002 (LY, 50µM) for one hour prior to infection. They were then infected with one of four different strains of GBS at an MOI of 10 for one hour, washed, and treated with antibiotics for an additional hour prior to lysate collection. Lysates were assessed for phosphorylated (active) p70s6k **(A, B)**, and densitometry was used to compare differences between infection conditions. Representative Western blots from one biological replicate with its corresponding loading control (beta actin) are shown **(A)**. Equal amounts of the same protein lysate preparations were loaded onto the gels for each protein. Densitometry values represent pooled results from at least three independent biological replicates, and error bars represent standard deviations of the mean **(B)**. Significant differences between DMSO and LY treatments for each condition were determined by t-test (*, p=0.01-0.05; **, p=0.001-0.01).

We then assessed whether activity of p70s6k was impacted by inhibition of the PI3K-Akt pathway during GBS infection using a well characterized inhibitor, LY294002 (LY) (Vlahos et al., 1994). For this analysis, we selected four GBS strains from our 15 strain panel, one from each ST represented. Our results indicated that the addition of LY did significantly reduce phospho-p70s6k levels in macrophages in response to all four GBS strains tested (**Figure 2**). These results indicated that LY would be an effective inhibitor for evaluating the role of the PI3K-Akt pathway in our infection system.

### Diverse GBS strains differentially impact actin cytoskeleton rearrangements in macrophages

**Figure 3:**
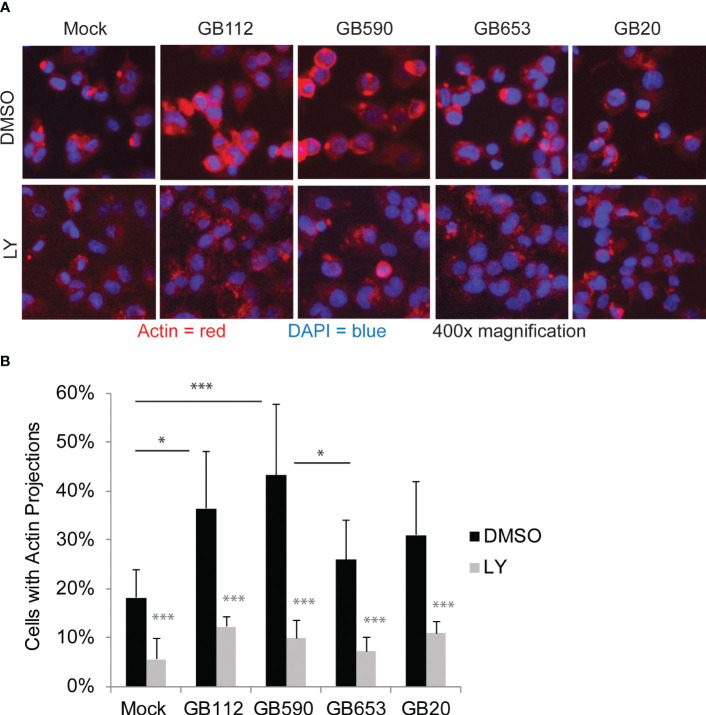
GBS induces the formation of actin projections in macrophages. THP-1 macrophages were treated with DMSO or LY294002 (LY, 50µM) for one hour prior to infection. They were then infected with one of four different strains of GBS at an MOI of 10 for one hour. Antibiotics were then applied for 1 hour to kill extracellular bacteria, and cells were washed, fixed, and prepared for immunofluorescence microscopy. β-actin (red) was visualized with Alexafluor594, and nuclei were stained with DAPI (blue) **(A)**. The percentage of cells with actin projections was determined for each condition **(B)**. At least three biological replicates were performed for each condition containing DMSO, and at least two biological replicates were performed for conditions containing LY, with at least three fields captured per replicate. At least 2,500 cells were counted per DMSO condition and at least 1,500 cells were counted per LY condition. Significance was determined by ANOVA (p=0.0005) and *post-hoc* Tukey’s tests to compare the DMSO conditions to each other (black asterisks), and error bars represent standard deviation of the mean. T-tests were performed to compare DMSO conditions to their corresponding LY conditions (gray asterisks). For both Tukey’s tests and t-tests, p<0.05 was considered statistically significant (*, p=0.01-0.05; ***, p=0.0001-0.001).

Having confirmed that a key target of the PI3K-Akt pathway was upregulated in response to certain GBS strains and that its levels could be readily manipulated with a PI3K-Akt pathway inhibitor, we next sought to link changes in the PI3K-Akt signaling pathway to specific outcomes in GBS-infected macrophages. As this pathway has been associated with phagocytic uptake of various microbes by macrophages (Maisey et al., 2008; Lovewell et al., 2014; Lv et al., 2019; Chang et al., 2020; Mu et al., 2020), we first assessed whether changes in the PI3K-Akt pathway were linked with rearrangements of the actin cytoskeleton and subsequent GBS uptake. We began with an initial visual analysis using immunofluorescence microscopy to determine whether four different strains of GBS from different STs induced the formation of actin projections in macrophages. The differentiated THP-1 cells were exposed to one of each of the four GBS isolates for 1 hour, washed, and incubated for an additional hour prior to fixation. Differences in the production of actin projections were visualized using antibodies specific for beta-actin (**Figure 3**), and DAPI was used to visualize THP-1 nuclei. Cell-associated GBS were also visible with DAPI staining (**Supplementary Figure 1**). Interestingly, both GB112 and GB590 (ST-17 and ST-19 isolates, respectively), which had induced the highest levels of phospho-p70s6k (**Figure 2**), induced a significantly greater number of actin projections in infected macrophages compared to mock infection (**Figure 3**). This result is consistent with previous findings from our group and our collaborators, indicating that strains from these STs induce greater levels of phagocytic uptake by macrophages than other STs (Korir et al., 2017a; Flaherty et al., 2021). When the THP-1 cells were incubated with LY294002 (LY) prior to infection, there was a significant decrease in the formation of actin projections compared to the corresponding vehicle control (DMSO) for each condition (**Figure 3**). Though the actin appeared to be present at similar levels to the corresponding DMSO conditions, it was more dispersed throughout the cytosol in the presence of LY (**Figure 3**). This supports the link between activation of the PI3K-Akt pathway and regulation of the actin cytoskeleton in our experimental system.

### The PI3K-Akt pathway regulates phagocytosis and GBS survival in macrophages

**Figure 4:**
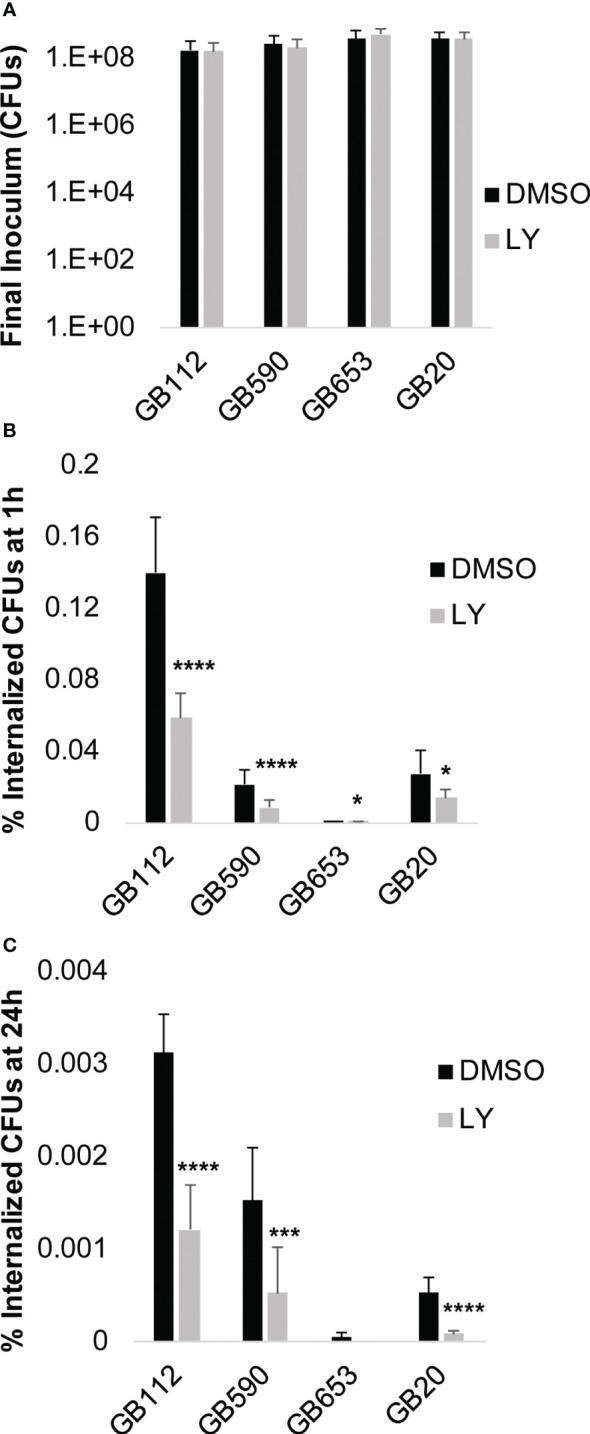
PI3K-Akt signaling promotes phagocytosis and survival of GBS. THP-1 cells were infected with GBS at an MOI of 10 for 1 hour in the presence or absence of LY294002 (LY, 50µM), a PI3K pathway inhibitor. **(A)** After 1 hour, a sample was collected from each biological replicate and plated to determine the final inoculum of GBS. **(B, C)** Antibiotics were added to kill extracellular bacteria, and cells were incubated for 1 hour to determine GBS uptake **(B)**, or 24 hours to assess long-term GBS survival within macrophages **(C)**. Internalized bacteria were collected and plated; CFUs were normalized to the final inoculum for each strain to determine the percent internalized CFUs at 1 hour or 24 hours **(B, C)**. At least six biological replicates were performed per condition. Significant differences between DMSO and LY for each condition were determined by t-test. Error bars represent the standard deviation of the mean; p-values <0.05 were considered significant (*, p=0.01-0.05; ***, p=0.0001-0.001; ****, p<0.0001).

To determine whether the PI3K-Akt pathway could be directly linked with these changes in the actin cytoskeleton and subsequent GBS uptake by macrophages, we next assessed phagocytic uptake following GBS infection in the presence of LY294002 (LY) compared to a vehicle control (DMSO). Macrophages were pre-treated with DMSO or LY for 1 hour, and then they were infected with GBS for 1 hour. At this point, a final inoculum was collected to compare bacterial levels across conditions (**Figure 4A**). Of note, there was not a statistical difference between DMSO *vs*. LY for any of the strains, indicating that the inhibitor did not impact bacterial viability directly for the conditions tested. The macrophages were then washed to remove extracellular bacteria and treated with antibiotics for an additional hour prior to collecting intracellular bacteria and quantifying them via colony counting assays (**Figure 4B**). Our results demonstrated that LY significantly reduced GBS uptake by macrophages for all four strains assessed, providing support for our hypothesis that GBS induction of PI3K-Akt signaling promotes phagocytic uptake (**Figure 4B**). In order to determine whether inhibition of the PI3K-Akt pathway also impacted the long-term survival of GBS within macrophages, we performed the same experiment to quantify intracellular GBS 24 hours after the initial infection period. Our results demonstrated that PI3K-Akt signaling promotes the intracellular survival of GBS, as the inhibitor (LY) significantly reduced the levels of viable intracellular GBS at this time point when compared to the vehicle control condition (DMSO) for the corresponding strain (**Figure 4C**).

### The PI3K-Akt pathway impacts macrophage survival following GBS infection

**Figure 5:**
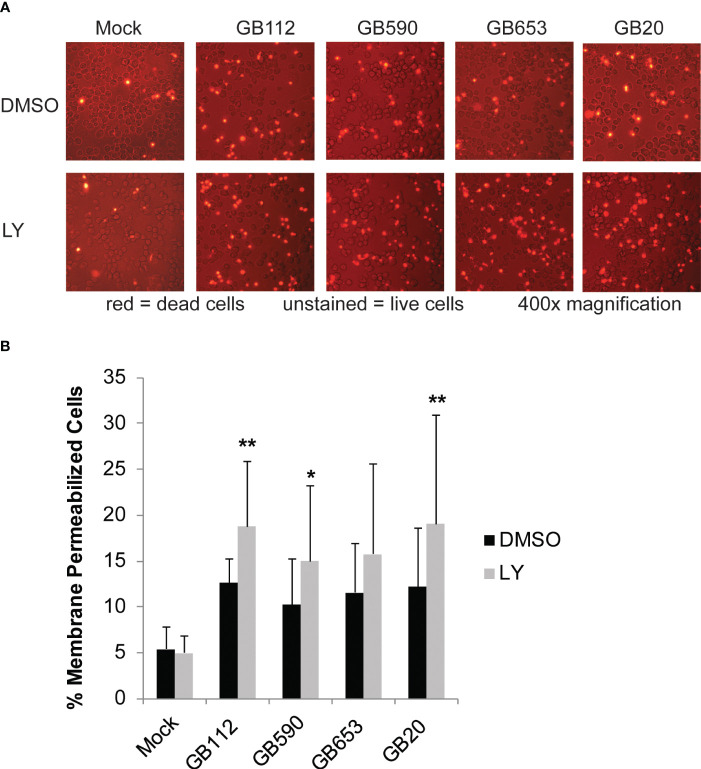
PI3K-Akt signaling reduces death in GBS-infected macrophages. THP-1 cells were infected with GBS at an MOI of 10 bacteria per host cell for 1 hour in the presence or absence of LY294002, a PI3K-Akt inhibitor (LY, 50µM). Cells were washed, treated with antibiotics, and incubated for an additional 24 hours. Dead cells were visualized by microscopy using a fluorescent dye, ethidium homodimer-1 (4µM in PBS). At least six biological replicates were performed per condition, with at least three fields captured per well; at least 9,000 cells were counted per condition. Representative microscopy images from each condition are shown **(A)**, and results from the 6 replicates were averaged and are graphed here **(B)**. Error bars represent standard deviations of the mean. Significant differences between DMSO and LY treatments for each condition were determined by t-test (*, p=0.01-0.05; **, p=0.001-0.01).

PI3K-Akt signaling has also been shown to have an important impact in eukaryotic cell survival in many contexts (Busca et al., 2014; Follo et al., 2015; Yu and Cui, 2016; Vergadi et al., 2017). Therefore, we next wanted to determine whether GBS manipulation of this pathway had any impact on the survival of the macrophages themselves. To determine whether there was a connection between PI3K-Akt pathway activation and macrophage survival, macrophages were pre-treated with DMSO or LY for 1 hour, infected with GBS for 1 hour, washed to remove extracellular bacteria, and then incubated in the presence of antibiotics for an additional 24 hours. An ethidium homodimer membrane permeabilization assay was then utilized to determine differences in macrophage cell death at 24 hours after the initial infection period. When the PI3K-Akt pathway was inhibited with LY, we observed a significant increase in macrophage cell death in response to three of the four strains analyzed, with a similar trend observed for the fourth strain (**Figure 5**). This indicates that the PI3K-Akt pathway aids in macrophage viability when macrophages are infected with GBS. Because activity of PI3K-Akt signaling appears to both promote macrophage survival as well as survival of intracellular GBS, we speculate that GBS may promote PI3K-Akt signaling in order to form an intracellular niche for continued persistence within the infected host.

As the type of cell death occurring within the infected macrophages could have important implications relating to the fate of the intracellular GBS and on the immune response to the infection, we next sought to determine the type of cell death being induced by GBS in the infected macrophages (**Supplementary Figures 2-6**). We selected caspase-1 as a marker of pyroptosis (**Figure S2**), caspase-3 as a marker of classic apoptosis (**Figure S3**), and phospho-MLKL as a marker of necroptosis (**Figure S4**), as these different forms of programmed cell death have been associated with many types of microbial infection (Ashida et al., 2011). We evaluated all three of these proteins by Western Blotting at both 24 and 48 hours post-infection. When we evaluated our infected cell lysates for the presence of full length and cleaved (activated) caspase-3 (**Figure S3**), we did not observe any significant changes in either full length or cleaved caspase-3 in response to infection with any of the strains analyzed at either 24 or 48 hours post-infection. This led us to conclude that classic apoptosis is unlikely to be the major form of cell death induced under these conditions.

When we assessed caspase-1, we observed increased production of full length caspase-1 as well as a potentially active high molecular weight form of caspase-1 (which we refer to in our figures as aggregated caspase-1) at 24 hours post-infection (**Figure S2**). We did not observe a detectable change in the usual activated form of caspase-1 at either time point in our cell lysates; cleaved caspase-1 is generally seen at a molecular weight of 10-20kDa (**Figure S2**). Of note, Shamaa et al. have reported that caspase-1 may be produced by macrophages in an active high molecular weight form (>200kDa) similar to what we observed in these studies, and that this form of active caspase-1 effectively activates IL-1β and tends to be significantly more stable than the 20kDa active form (Shamaa et al., 2015). Increases in high molecular weight caspase-1 were significant for the GB590, GB653, and GB20 strains at 24 hours, but not at 48 hours post-infection. Increases in full length caspase-1 were significant at 24 hours post-infection for the GB590 and GB20 infection conditions, but no significant differences were observed at 48 hours (**Figure S2**). Consistent with the possibility that caspase-1 plays a role in GBS-induced macrophage death in our experimental system, we also observed that cell death could be significantly reduced at the 48 hour time point, albeit modestly, by treating the THP-1 cells with the pan-caspase inhibitor Z-VAD-fmk prior to infection with GBS (**Figure S6**). Curiously, this effect was not yet apparent at the 24 hour time point. Furthermore, we have previously observed significant production of IL-1β from GBS-infected macrophages 24 hours post-infection, which would also be consistent with induction of pyroptosis (Flaherty et al., 2019a).

Finally, we assessed the cells for the presence of phospho-MLKL as a marker of necroptosis (**Figure S4**). Here, we observed a trend toward increased phospho-MLKL in response to several of the strains at both 24 hours and 48 hours post-infection. This increase was significant for the GB112 and GB590 strains at 24 hours post-infection and for the GB20 strain at 48 hours post-infection (**Figure S4**). Despite the increase in this key necroptosis marker, we did not observe a decrease in cell death when the cells were treated with the necroptosis inhibitor Necrostatin-1 at the 24 or 48 hour time points for any of the strains except the GB112 (ST-17) condition (data not shown). For this reason, we speculate that pyroptosis may be the primary form of cell death occurring in the infected cells, though it is possible that multiple forms of cell death are being induced, particularly by strains with enhanced virulence properties, like the GB112 strain. Both pyroptosis and necroptosis fall under the umbrella of programmed necrosis, which would also be consistent with our visual observations that the infected THP-1 cells exhibit a swollen and somewhat translucent appearance compared to uninfected cells when viewed with simple live cell imaging 24 hours post-infection (**Figure S5**).

### PI3K-Akt signaling promotes activation of the NF-kappa-B signaling pathway in GBS-infected macrophages

**Figure 6:**
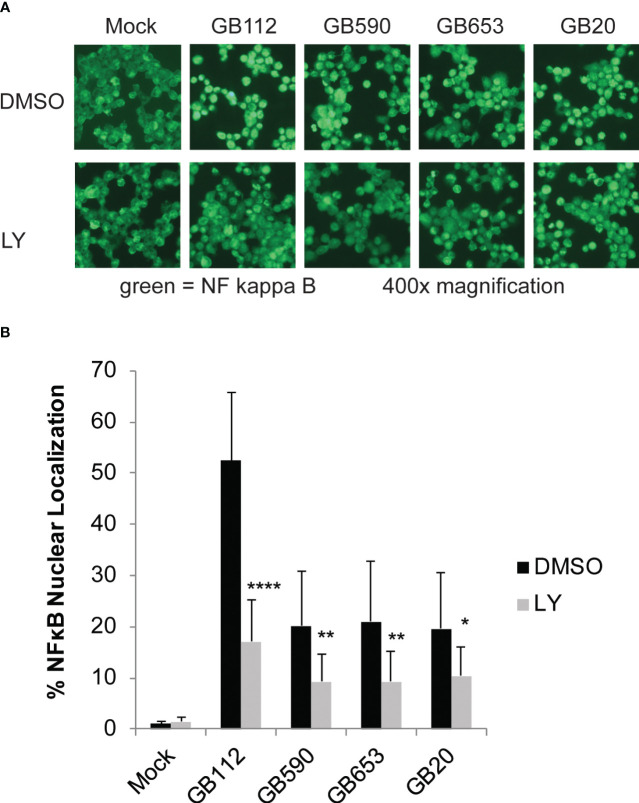
PI3K-Akt signaling promotes activation of the NF-kappa-B signaling pathway in GBS-infected macrophages. THP-1 cells were infected with GBS at an MOI of 10 bacteria per host cell for 1 hour in the presence or absence of LY294002, a PI3K-Akt inhibitor (LY, 50µM). The cells were then washed and treated with antibiotics for an additional hour prior to fixation, nuclear staining (DAPI), and detection of NF-κB p65 (Alexa Fluor 488) by immunofluorescence microscopy. **(A)** One representative field visualizing NF-κB p65 (Alexa Fluor 488) is shown for each condition. The percentage of NF-κB nuclear localization compares the number of cells with positive nuclear localization (Alexa Fluor 488) to the total cell number in a given field (DAPI) using ImageJ. **(B)** Results from three independent biological replicates were obtained for each condition. For each biological replicate of each condition, at least three separate fields were captured to obtain a minimum of 5,500 cells. The average percent nuclear localization was graphed for each condition, and error bars represent standard deviation of the mean. Significant differences between DMSO and LY treatments for each condition were determined by t-test (*, p=0.01-0.05; **, p=0.001-0.01; ****, p<0.0001).

One common downstream target of PI3K-Akt signaling is the NF-kappa-B pathway, which is known to be associated both with the regulation of cell survival as well as the induction of inflammatory signaling in a variety of contexts (Tsatsanis et al., 2006; Vandenabeele et al., 2010; Newton and Dixit, 2012; Lappas, 2013; Busca et al., 2014). We have previously demonstrated that this pathway is activated in response to GBS and that it is linked to the production of several inflammatory cytokines in response to GBS infection (Flaherty et al., 2019a; Flaherty et al., 2021). We next sought to determine whether the PI3K-Akt pathway is an upstream contributor to the activation of this pathway during infection. Macrophages were pre-treated with either DMSO or LY as before, infected with GBS for 1 hour, washed to remove extracellular bacteria, and treated with antibiotics for an additional hour prior to fixing cells for microscopy. Inhibition of PI3K-Akt signaling was shown to reduce NF-kappa-B nuclear localization in response to all four strains analyzed (**Figure 6**). This indicates that the PI3K-Akt pathway does, indeed, contribute to NF-kappa-B activation in response to GBS infection in macrophages.

## Discussion

Overall, the goal of this study was to explore the role of the PI3K-Akt pathway in GBS-infected macrophages and to assess the implications of strain-dependent differences in its activation. This investigation stemmed from prior work in which we had observed activation of several PI3K-Akt pathway members during GBS infection of THP-1 macrophages by antibody array and also separately observed strain-dependent differences in phagocytic uptake and long-term survival of GBS within macrophages (Korir et al., 2017a; Flaherty et al., 2021). In these prior studies we detected small intracellular populations of GBS within the THP-1 macrophages following infection with all 15 of the GBS strains used in this study (Flaherty et al., 2021). As an initial step to determine whether there was a link between these two observations, we examined the formation of actin projections in macrophages that had been exposed to four different strains of GBS from diverse sequence types. We observed that actin projections were more prevalent in macrophages when they were infected with ST-17 and ST-19 strains (GB112 and GB590, respectively), both of which are capsule type III strains, compared to other GBS strains tested. We interpreted these rearrangements of the actin cytoskeleton to be indicative of increased phagocytic uptake, which would be consistent with the observation that differentiated THP-1 cells engulf greater numbers of ST-17 and ST-19 strains compared to other STs (Korir et al., 2017a; Flaherty et al., 2021). The formation of these actin projections was largely inhibited by treatment with a PI3K-Akt pathway inhibitor, LY294002, prior to infection. This observation is consistent with other studies that have shown that the use of PI3K inhibitors, including LY294002 and wortmannin, can have dramatic effects on downstream members of the PI3K pathway, actin polymerization, and phagocytosis in macrophages in response to other bacterial pathogens, such as *Helicobacteri pylorii* (Allen et al., 2005). Similarly, this work supports the results of various reports demonstrating that GBS can induce PI3K-Akt-dependent actin cytoskeleton rearrangements to enter a variety of cell types, such as human endometrial cells, epithelial cells, fibroblasts, and human endothelial cells (Tyrrell et al., 2002; Burnham et al., 2007; Goluszko et al., 2008; de Oliveira et al., 2018). It would be interesting to evaluate whether uptake via the PI3K-Akt pathway in these other cell types also varies based on sequence and capsule types in more detail.

Of note, GBS strains that induced more actin projections in infected macrophages at the one hour time point also tended to exhibit higher intracellular GBS viability at the 24 hour time point as well. Our data demonstrating that treatment with a PI3K-Akt pathway inhibitor reduces both phagocytic uptake of GBS one hour post-infection as well as intracellular survival of GBS 24 hours post-infection support the hypothesis that the PI3K-Akt pathway contributes to the initial uptake and intracellular survival of GBS within macrophages, though the precise mechanisms involved require more exploration. This role seems to be more pronounced when macrophages are infected with the ST-17 and ST-19 strains (both CPS III), as these strains tend to activate the PI3K-Akt pathway to a greater extent than the other strains that were tested. Thus, while GBS can be included in the ranks of the many other pathogens that cleverly manipulate the host cytoskeleton to gain multiple benefits relating to colonization, uptake, and survival, the extent to which this strategy is employed seems to be somewhat strain-dependent (Tyrrell et al., 2002; Fettucciari et al., 2011).

In these studies, we did not seek to identify which bacterial virulence factors were responsible for the strain-dependent differences in phagocytic uptake and intracellular survival, but our findings were in line with other studies that have investigated these mechanisms in more detail. The PI3K-Akt pathway has been shown to be regulated in response to other intracellular pathogens such as *Mycobacterium tuberculosis, Francisella tularensis, Salmonella*, and *Leishmania*, resulting in a reduced inflammatory cytokine response, inhibition of the classic apoptosis pathway through several mechanisms, and in some cases, inhibition of the phagosome maturation process, which can allow for prolonged persistence of these microbes intracellularly (Ruhland et al., 2007; Harding and Boom, 2010; Medina et al., 2010; Thi et al., 2012). Korir et al. utilized a multiple stress medium to evaluate the ability of 30 different GBS strains of varying STs to survive phagosomal stressors such as acidic pH, hydrogen peroxide, nitric oxide, lysozyme, and cupric chloride, and found that CPSIII strains such as those in the ST-17 group were best equipped to survive within macrophages following phagocytosis (Korir et al., 2017a). Though much is still unknown regarding which virulence genes GBS uses to survive in macrophages following phagocytosis, genes such as *ponA, cylE*, *sodA*, and the *CovR/S 2* component regulatory system have all been found to enhance the ability of GBS to survive inside phagosomes and withstand macrophage defenses such as oxidative stress (Poyart et al., 2001; Liu et al., 2004; Hamilton et al., 2006; Cumley et al., 2012; Korir et al., 2017a).

In addition to aiding in the regulation of bacterial uptake and subsequent intracellular survival of bacteria, other studies have demonstrated that rearrangements of the cytoskeleton by pathogens can also impact signaling pathways related to the viability of the host cell (Liu et al., 2001; Ahmadian et al., 2002; Fiorentini et al., 2003; Bhavsar et al., 2007; Labbé and Saleh, 2008; Fettucciari et al., 2011; Owen et al., 2014). Such reports are consistent with our observation that the PI3K-Akt pathway promotes macrophage survival during GBS infection, as there was an increase in macrophage cell death when this pathway was inhibited with a small molecule inhibitor (LY). As we did not observe evidence of the classic apoptosis marker caspase-3, but rather detected enhanced levels of two different proteins involved in forms of programmed necrosis (caspase-1 and phospho-MLKL), we believe the infected macrophages are dying primarily from a form of programmed necrosis. Because cell death could also be reduced with the use of caspase inhibitors, we hypothesize that pyroptosis, which relies on caspase-1 activation, is the predominant mechanism involved.

This finding may have important implications relating to the generation of an intracellular niche for GBS. It is possible that the reduction in GBS intracellular survival in the presence of the PI3K-Akt pathway inhibitor can be explained, at least in part, by the fact that more of the macrophages are dying when this pathway is inhibited. Macrophage death would thereby destroy the safe haven being utilized by this small population of intracellular bacteria that seem to be able to avoid or delay being killed by the macrophages following uptake. If this is, indeed, the case, such a relationship could influence outcomes such as GBS dissemination across tissue barriers and GBS resistance to treatment with antibiotics.

As cell death signaling and stimulation of inflammatory cascades are often linked, we also explored the relationship between the PI3K-Akt pathway and the induction of inflammatory signaling through the NFκB signaling pathway. Treatment with LY significantly reduced NFκB nuclear localization in infected macrophages, indicating that PI3K-Akt signaling does contribute to activation of this key inflammatory mediator in response to GBS infection. Though the PI3K-Akt pathway can also serve as a negative regulator of NFκB signaling in response to many stimuli, PI3K-Akt pathway-driven activation of NFκB (similar to what we observed in these studies) has been observed in macrophages infected with other intracellular pathogens such as *Mycobacterium bovis* and HIV, or following exposure to purified bacterial components such as lipopolysaccharide (LPS) (Reddy et al., 1997; Méndez-Samperio et al., 2009; Liu et al., 2014; Zha et al., 2014; Vergadi et al., 2017). However, as NFκB is such a central player in the inflammatory response, we expect that additional upstream mediators also contribute to its activation in response to GBS infection.

**Figure 7:**
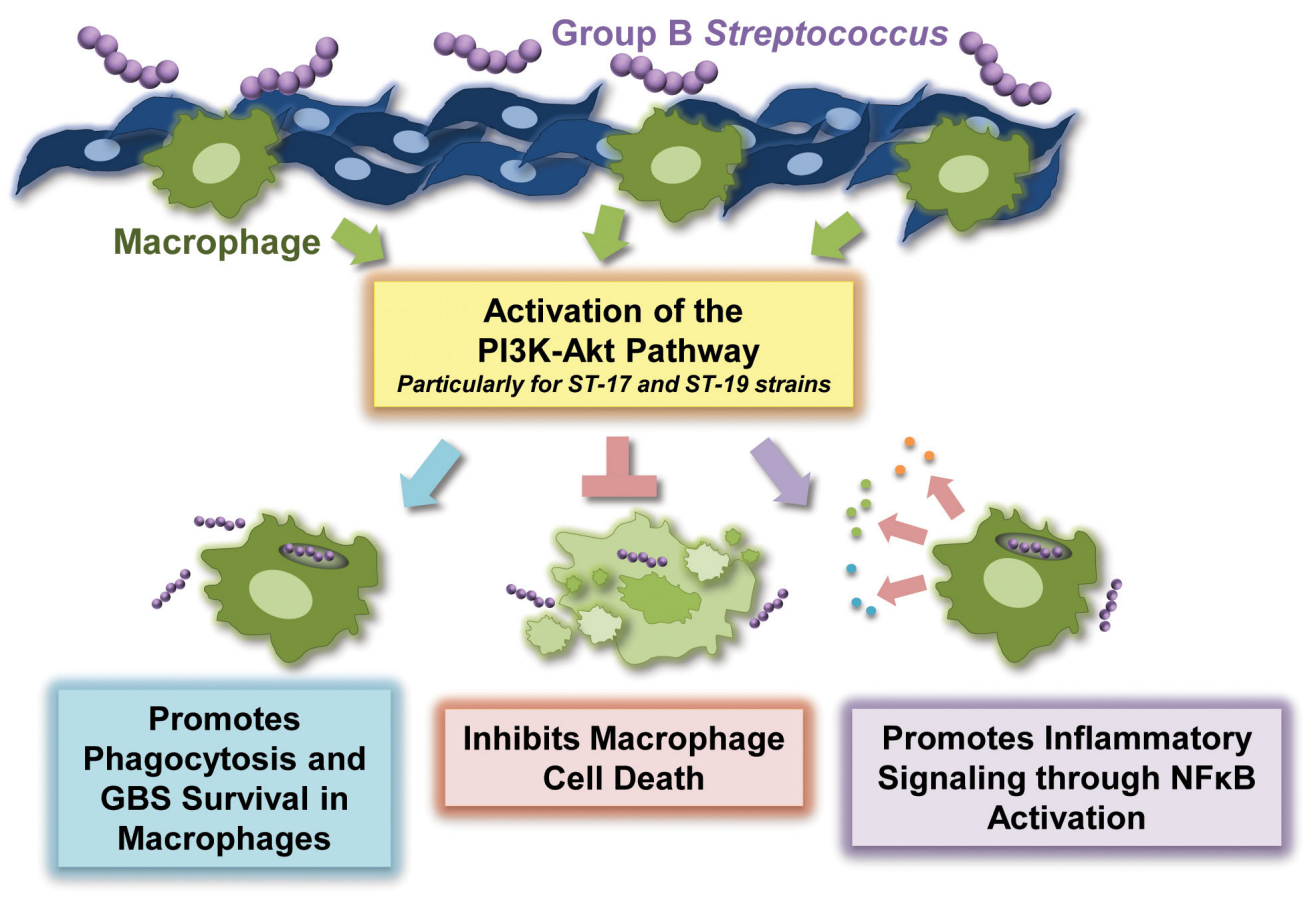
Summary of the role of the PI3K-Akt pathway in the regulation of the macrophage response to Group B Streptococcus (GBS).

In summary, our observations indicate that the PI3K-Akt pathway contributes to several important aspects of GBS infection in macrophages, including phagocytic uptake, intracellular survival of GBS, survival of GBS-infected macrophages, and the induction of inflammatory signaling cascades (**Figure 7**). Strain-dependent differences in outcomes such as phagocytic uptake and intensity of inflammatory signaling may be at least partially explained by the fact that certain STs and CPS types differentially activate this key signaling pathway. Thus, our findings strengthen the growing body of evidence supporting the notion that genetically diverse GBS strains induce key host signaling cascades to varying extents, which likely contributes to critical differences in pathology (Fettucciari et al., 2011; Korir et al., 2014; Korir et al., 2017a; Flaherty et al., 2019a; Flaherty et al., 2019b; Flaherty et al., 2021). With continued careful exploration, we may be able to identify ways in which we can inhibit or otherwise manipulate the PI3K-Akt pathway in order to lessen disease severity during infection with more virulent GBS strains.

## Data availability statement

The raw data supporting the conclusions of this article will be made available by the authors, without undue reservation.

## Ethics statement

Ethical approval was not required for the studies on animals in accordance with the local legislation and institutional requirements because only commercially available established cell lines were used.

## Author contributions

RF: Conceptualization, Formal Analysis, Funding acquisition, Investigation, Methodology, Supervision, Writing – review & editing, Project administration, Resources, Validation, Visualization. YD: Formal Analysis, Investigation, Writing – original draft, Visualization. MT: Formal Analysis, Investigation, Writing – original draft, Visualization. JK: Formal Analysis, Investigation, Visualization, Writing – original draft.

## Supplementary material

Supplementary Figure 1 GBS are visible associated with THP-1 macrophages following infection. THP-1 cells were infected with GBS at an MOI of 10 bacteria per host cell for 1 hour. The cells were then washed and treated with antibiotics for an additional hour prior to fixation, nuclear staining (DAPI), and detection of actin (AlexaFluor594) by immunofluorescence microscopy. One representative field per condition from the experiment shown in **Figure 3** has been provided to allow for visualization of GBS at a high enough intensity for the bacteria to be seen associated with the macrophages.

Supplementary Figure 2 GBS influences Caspase-1 production and activity in infected macrophages. THP-1 macrophages were infected with one of four different strains of GBS at an MOI of 10 for one hour, washed, and treated with antibiotics for an additional 24-48 hours prior to lysate collection. Soluble lysate fractions were assessed for full length, cleaved, or high molecular weight versions (aggregated) of caspase-1, a key regulator of pyroptosis **(A, B)**; densitometry was used to compare differences between infection conditions for each of these forms of caspase-1. Representative Western blots from one biological replicate with its corresponding loading control (beta-actin or beta-tubulin) are shown **(A)**. Equal amounts of the same protein lysate preparations were loaded onto the gels for each protein. Densitometry values represent pooled results from at least three independent biological replicates, and error bars represent standard deviations of the mean **(B)**. Significant differences between mock infection and GBS infection conditions were determined by ANOVA, followed by *post-hoc* Dunnett’s testing (*, p=0.01-0.05; **, p=0.001-0.01; ***, p=0.0001-0.001; ****, p<0.0001).

Supplementary Figure 3 GBS does not impact Caspase-3 production and activity in macrophages under the experimental conditions analyzed. THP-1 macrophages were infected with one of four different strains of GBS at an MOI of 10 for one hour, washed, and treated with antibiotics for an additional 24-48 hours prior to lysate collection. Soluble lysate fractions were assessed for full length or cleaved versions of caspase-3, a key regulator of classic apoptosis **(A, B)**; densitometry was used to compare differences between infection conditions for both of these forms of caspase-3. Representative Western blots from one biological replicate with its corresponding loading control (beta-actin) are shown **(A)**. Equal amounts of the same protein lysate preparations were loaded onto the gels for each protein. Densitometry values represent pooled results from at least three independent biological replicates, and error bars represent standard deviations of the mean **(B)**. Significant differences between mock infection and GBS infection conditions were determined by ANOVA, followed by *post-hoc* Dunnett’s testing (*, p=0.01-0.05; **, p=0.001-0.01; ***, p=0.0001-0.001; ****, p<0.0001).

Supplementary Figure 4 GBS influences phospho-MLKL activity following infection in macrophages. THP-1 macrophages were infected with one of four different strains of GBS at an MOI of 10 for one hour, washed, and treated with antibiotics for an additional 24-48 hours prior to lysate collection. Soluble lysate fractions were assessed for phosphorylated MLKL, a key regulator of necroptosis **(A, B)**; densitometry was used to compare differences in phospho-MLKL between infection conditions. Representative Western blots from one biological replicate with its corresponding loading control (beta-actin) are shown **(A)**. Equal amounts of the same protein lysate preparations were loaded onto the gels for each protein. Densitometry values represent pooled results from at least three independent biological replicates, and error bars represent standard deviations of the mean **(B)**. Significant differences between mock infection and GBS infection conditions were determined by ANOVA, followed by *post-hoc* Dunnett’s testing (*, p=0.01-0.05; **, p=0.001-0.01; ***, p=0.0001-0.001; ****, p<0.0001).

Supplementary Figure 5 GBS infection induces cellular swelling in macrophages. THP-1 macrophages were infected with one of four different strains of GBS at an MOI of 10 for one hour, washed, and treated with antibiotics for an additional 24 hours prior to imaging the live, unstained cells with bright field microscopy. Representative images of the infected cells were collected using the 60x objective.

Supplementary Figure 6 Caspase inhibition reduces death in GBS-infected macrophages. THP-1 cells were infected with GBS at an MOI of 10 bacteria per host cell for 1 hour in the presence or absence of Z-VAD-fmk, a pan-caspase inhibitor (50µM). Cells were washed, treated with antibiotics, and incubated for an additional 24-48 hours. Dead cells were visualized by microscopy using a fluorescent dye, ethidium homodimer-1 (4µM in PBS). At least three biological replicates were performed per condition, with at least three fields captured per well. Cell counts were averaged and are graphed here for the 24 hour **(A)** and 48 hour **(B)** time points. Error bars represent standard deviations of the mean. Significant differences between DMSO and inhibitor treatments for each condition were determined by t-test (*, p=0.01-0.05; **, p=0.001-0.01; ***, p=0.0001-0.001; ****, p<0.0001).
