# Supplementary figures and images for: The PI3K-Akt pathway is a multifaceted regulator of the macrophage response to diverse group B Streptococcus isolates
Source: Front Cell Infect Microbiol. 2023 Oct 19;13:1258275. doi: 10.3389/fcimb.2023.1258275 (PMC10622663; doi:10.3389/fcimb.2023.1258275)

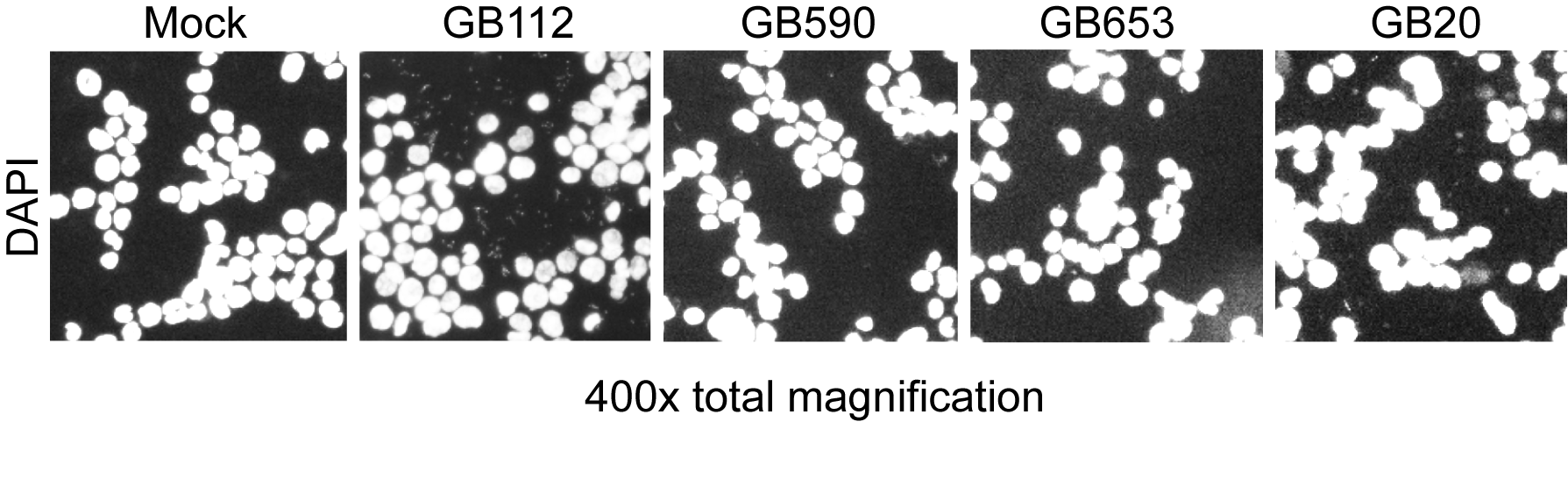

Supplement: Supplementary Figure 1 — GBS are visible associated with THP-1 macrophages following infection. THP-1 cells were infected with GBS at an MOI of 10 bacteria per host cell for 1 hour. The cells were then washed and treated with antibiotics for an additional hour prior to fixation, nuclear staining (DAPI), and detection of actin (AlexaFluor594) by immunofluorescence microscopy. One representative field per condition from the experiment shown in Figure 3 has been provided to allow for visualization of GBS at a high enough intensity for the bacteria to be seen associated with the macrophages. [file Image_1.tif]

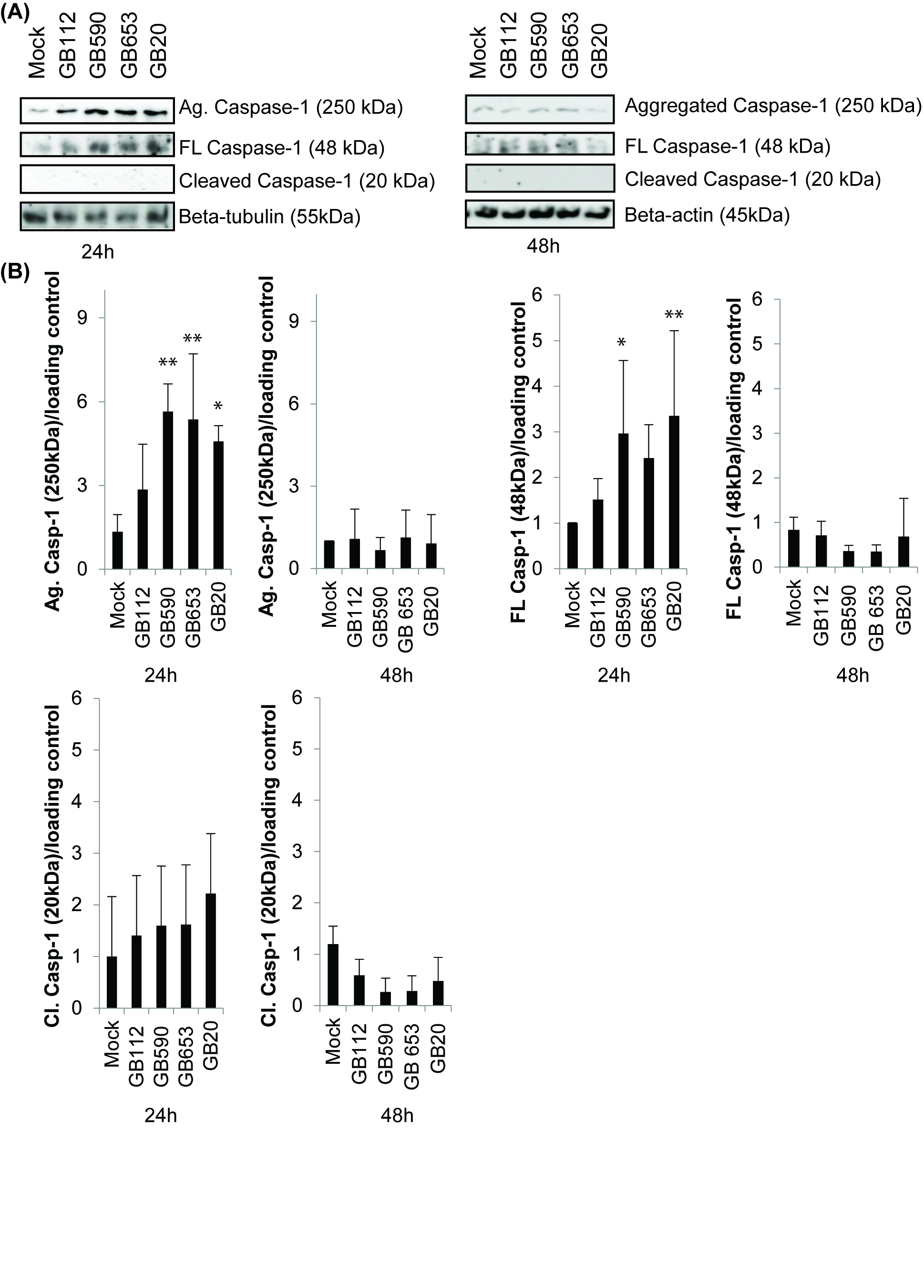

Supplement: Supplementary Figure 2 — GBS influences Caspase-1 production and activity in infected macrophages. THP-1 macrophages were infected with one of four different strains of GBS at an MOI of 10 for one hour, washed, and treated with antibiotics for an additional 24-48 hours prior to lysate collection. Soluble lysate fractions were assessed for full length, cleaved, or high molecular weight versions (aggregated) of caspase-1, a key regulator of pyroptosis (A, B); densitometry was used to compare differences between infection conditions for each of these forms of caspase-1. Representative Western blots from one biological replicate with its corresponding loading control (beta-actin or beta-tubulin) are shown (A). Equal amounts of the same protein lysate preparations were loaded onto the gels for each protein. Densitometry values represent pooled results from at least three independent biological replicates, and error bars represent standard deviations of the mean (B). Significant differences between mock infection and GBS infection conditions were determined by ANOVA, followed by post-hoc Dunnett’s testing (*, p=0.01-0.05; **, p=0.001-0.01; ***, p=0.0001-0.001; ****, p<0.0001). [file Image_2.tif]

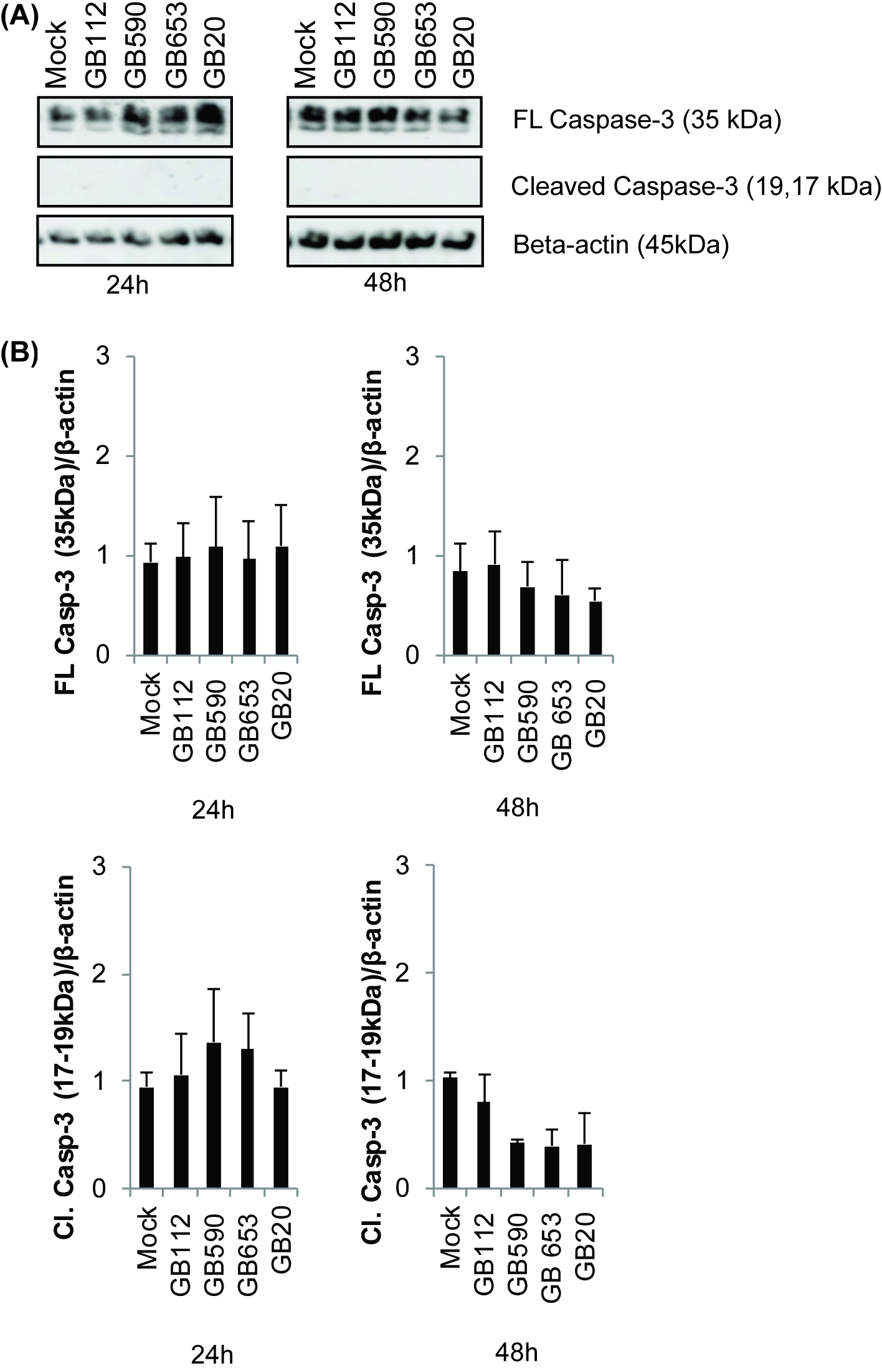

Supplement: Supplementary Figure 3 — GBS does not impact Caspase-3 production and activity in macrophages under the experimental conditions analyzed. THP-1 macrophages were infected with one of four different strains of GBS at an MOI of 10 for one hour, washed, and treated with antibiotics for an additional 24-48 hours prior to lysate collection. Soluble lysate fractions were assessed for full length or cleaved versions of caspase-3, a key regulator of classic apoptosis (A, B); densitometry was used to compare differences between infection conditions for both of these forms of caspase-3. Representative Western blots from one biological replicate with its corresponding loading control (beta-actin) are shown (A). Equal amounts of the same protein lysate preparations were loaded onto the gels for each protein. Densitometry values represent pooled results from at least three independent biological replicates, and error bars represent standard deviations of the mean (B). Significant differences between mock infection and GBS infection conditions were determined by ANOVA, followed by post-hoc Dunnett’s testing (*, p=0.01-0.05; **, p=0.001-0.01; ***, p=0.0001-0.001; ****, p<0.0001). [file Image_3.tif]

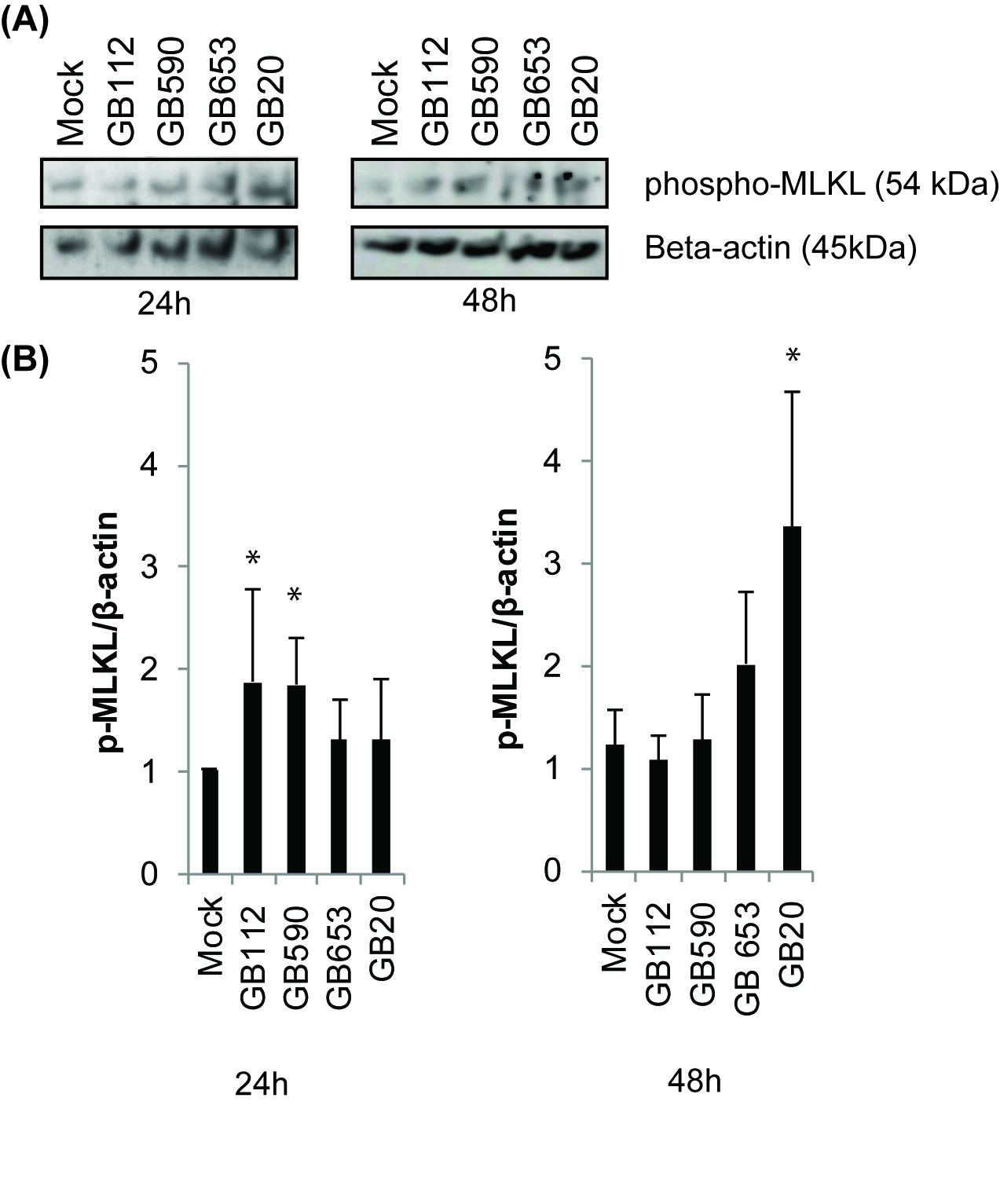

Supplement: Supplementary Figure 4 — GBS influences phospho-MLKL activity following infection in macrophages. THP-1 macrophages were infected with one of four different strains of GBS at an MOI of 10 for one hour, washed, and treated with antibiotics for an additional 24-48 hours prior to lysate collection. Soluble lysate fractions were assessed for phosphorylated MLKL, a key regulator of necroptosis (A, B); densitometry was used to compare differences in phospho-MLKL between infection conditions. Representative Western blots from one biological replicate with its corresponding loading control (beta-actin) are shown (A). Equal amounts of the same protein lysate preparations were loaded onto the gels for each protein. Densitometry values represent pooled results from at least three independent biological replicates, and error bars represent standard deviations of the mean (B). Significant differences between mock infection and GBS infection conditions were determined by ANOVA, followed by post-hoc Dunnett’s testing (*, p=0.01-0.05; **, p=0.001-0.01; ***, p=0.0001-0.001; ****, p<0.0001). [file Image_4.tif]

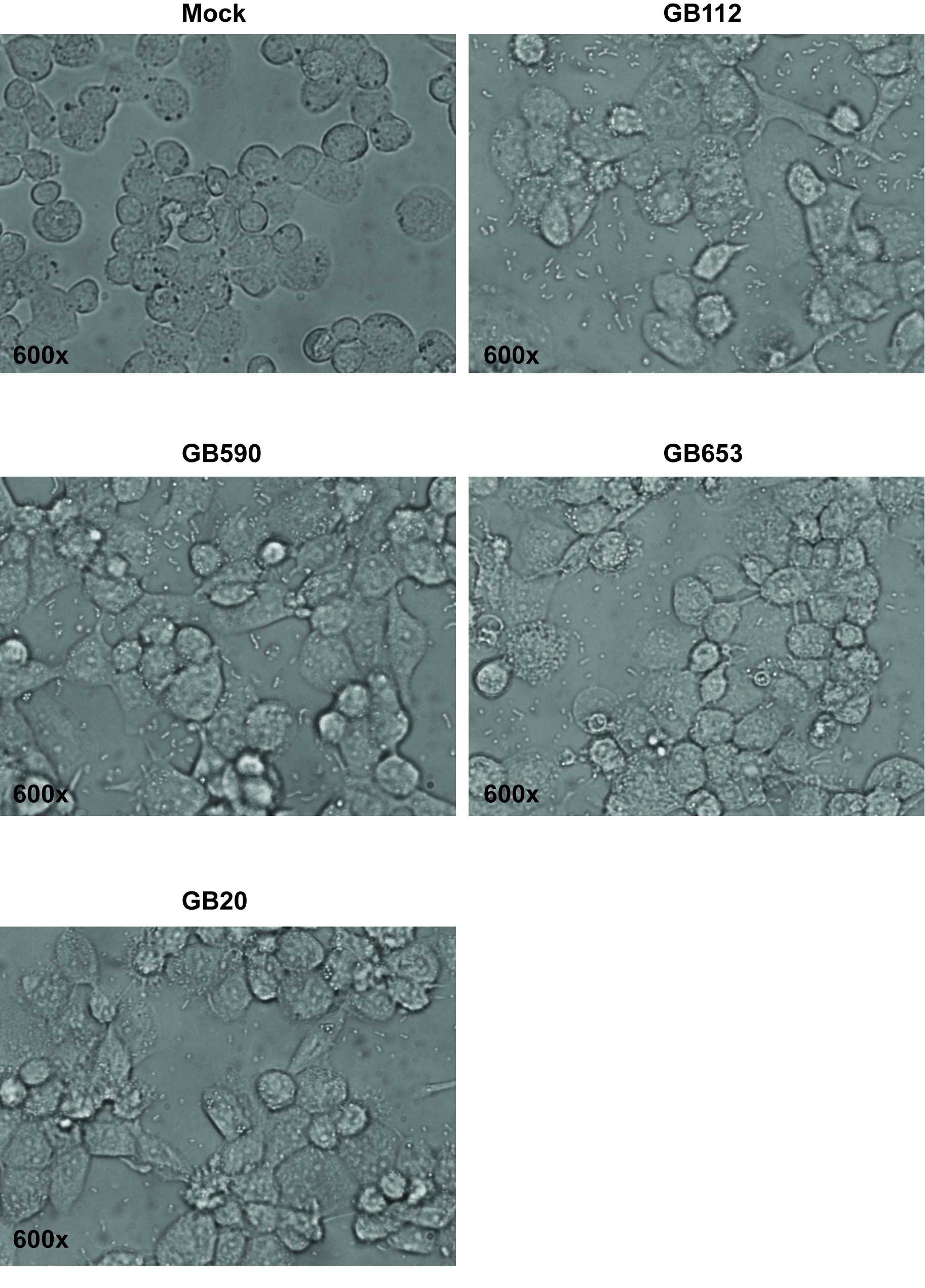

Supplement: Supplementary Figure 5 — GBS infection induces cellular swelling in macrophages. THP-1 macrophages were infected with one of four different strains of GBS at an MOI of 10 for one hour, washed, and treated with antibiotics for an additional 24 hours prior to imaging the live, unstained cells with bright field microscopy. Representative images of the infected cells were collected using the 60x objective. [file Image_5.tif]

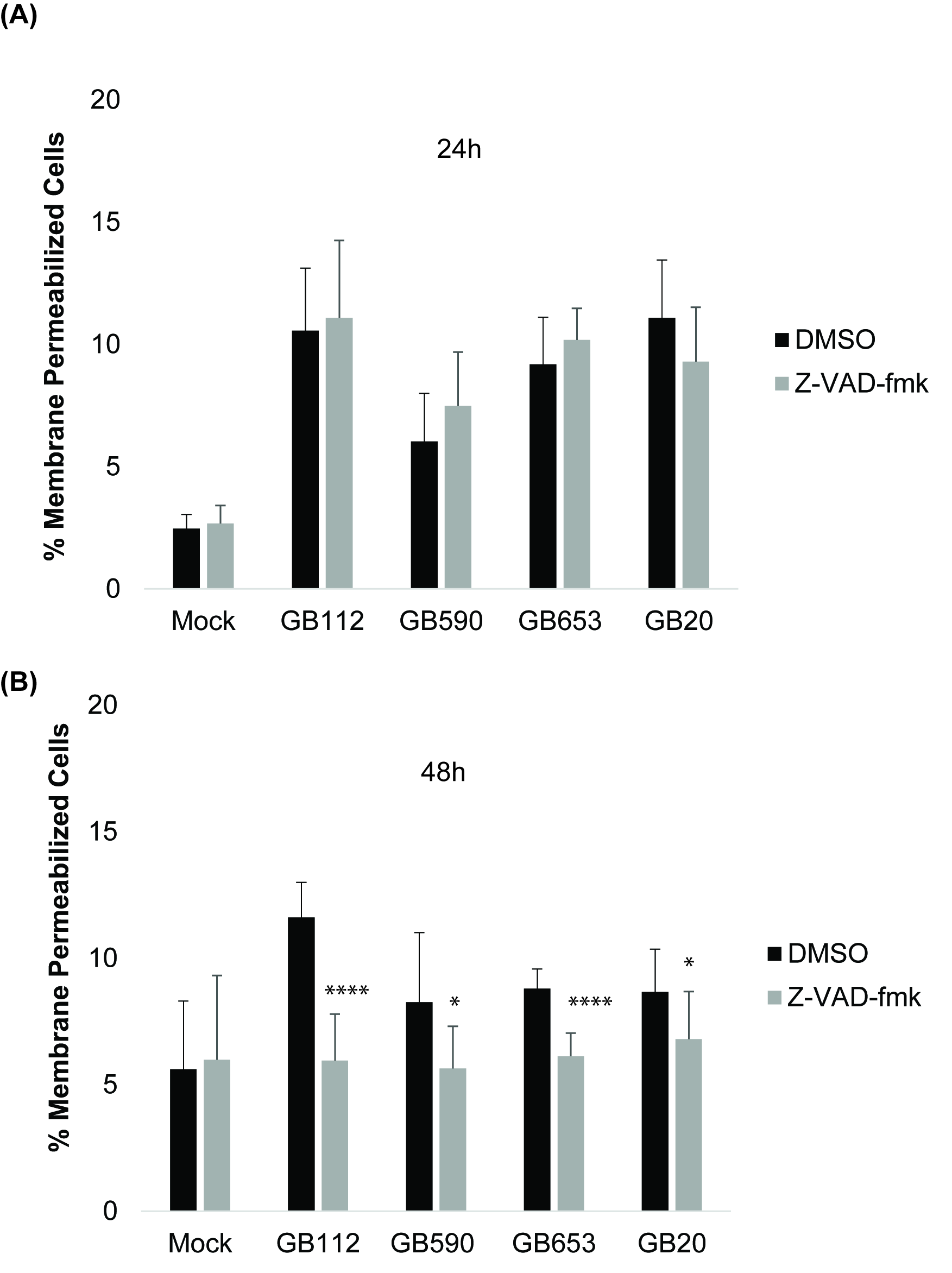

Supplement: Supplementary Figure 6 — Caspase inhibition reduces death in GBS-infected macrophages. THP-1 cells were infected with GBS at an MOI of 10 bacteria per host cell for 1 hour in the presence or absence of Z-VAD-fmk, a pan-caspase inhibitor (50µM). Cells were washed, treated with antibiotics, and incubated for an additional 24-48 hours. Dead cells were visualized by microscopy using a fluorescent dye, ethidium homodimer-1 (4µM in PBS). At least three biological replicates were performed per condition, with at least three fields captured per well. Cell counts were averaged and are graphed here for the 24 hour (A) and 48 hour (B) time points. Error bars represent standard deviations of the mean. Significant differences between DMSO and inhibitor treatments for each condition were determined by t-test (*, p=0.01-0.05; **, p=0.001-0.01; ***, p=0.0001-0.001; ****, p<0.0001). [file Image_6.tif]
